# Cross-model metabolomic signatures of dietary anti-inflammatory (poly) phenols in nutraceutical development: a systematic review

**DOI:** 10.3389/fmolb.2026.1824027

**Published:** 2026-10-08

**Authors:** Adesoji Fayomi, Emmanuel Adom, Alberta N. A. Aryee

**Affiliations:** 1 Department of Biochemistry, Ahmadu Bello University, Zaria, Nigeria; 2 Department of Pharmacognosy and Herbal Medicine, School of Pharmacy and Pharmaceutical Sciences, University for Development Studies, Tamale, Ghana; 3 Department of Human Ecology, Food Science & Biotechnology Program, College of Agriculture, Science and Technology, Delaware State University, Dover, DE, United States

**Keywords:** anti-inflammatory properties, dietary (poly) phenols, gut microbiome, metabolomics, nutraceuticals

## Abstract

Dietary (poly) phenols play a key role in nutraceutical development, due to their diverse positive health benefits such as antioxidant and anti-inflammatory properties. Although these bioactivities have been widely reported, the consistency of metabolomic signatures in experimental systems, ranging from *in vitro* models to human studies, remains unclear. This systematic review examined metabolomic responses to dietary anti-inflammatory (poly) phenols *in vitro*, animal, human, and mixed-model studies. Searches were conducted in PubMed, Scopus, Web of Science, and Google Scholar for studies published between 2020 and 2025. Seventy-eight eligible primary studies were included and synthesized narratively due to differences in interventions, models, sample matrices, analytical platforms, and outcomes. The synthesis showed model-dependent recurrence of several metabolomic signatures, with lipids, amino acids, and energy metabolism widely represented in the model groups, including phospholipids, sphingolipids, acylcarnitines, branched-chain and aromatic amino acids, kynurenine metabolites, TCA cycle intermediates, glycolytic metabolites, ketone bodies, and ATP markers. Changes in short-chain fatty acids were most consistent in animal and mixed-model gut studies but limited and matrix-dependent in human studies. Urolithins and ellagic acid-derived metabolites were most prominent in human and mixed-model studies, especially urinary biomarkers and microbiome-dependent metabolites. Overall, dietary anti-inflammatory (poly) phenols were associated with recurring metabolomic changes; however, these patterns were influenced by the model system, sample matrix, analytical platform, and intervention type. Further human studies are required to validate these candidate biomarkers and pathways for nutraceutical application.

## Graphical Abstract

**Figure:**
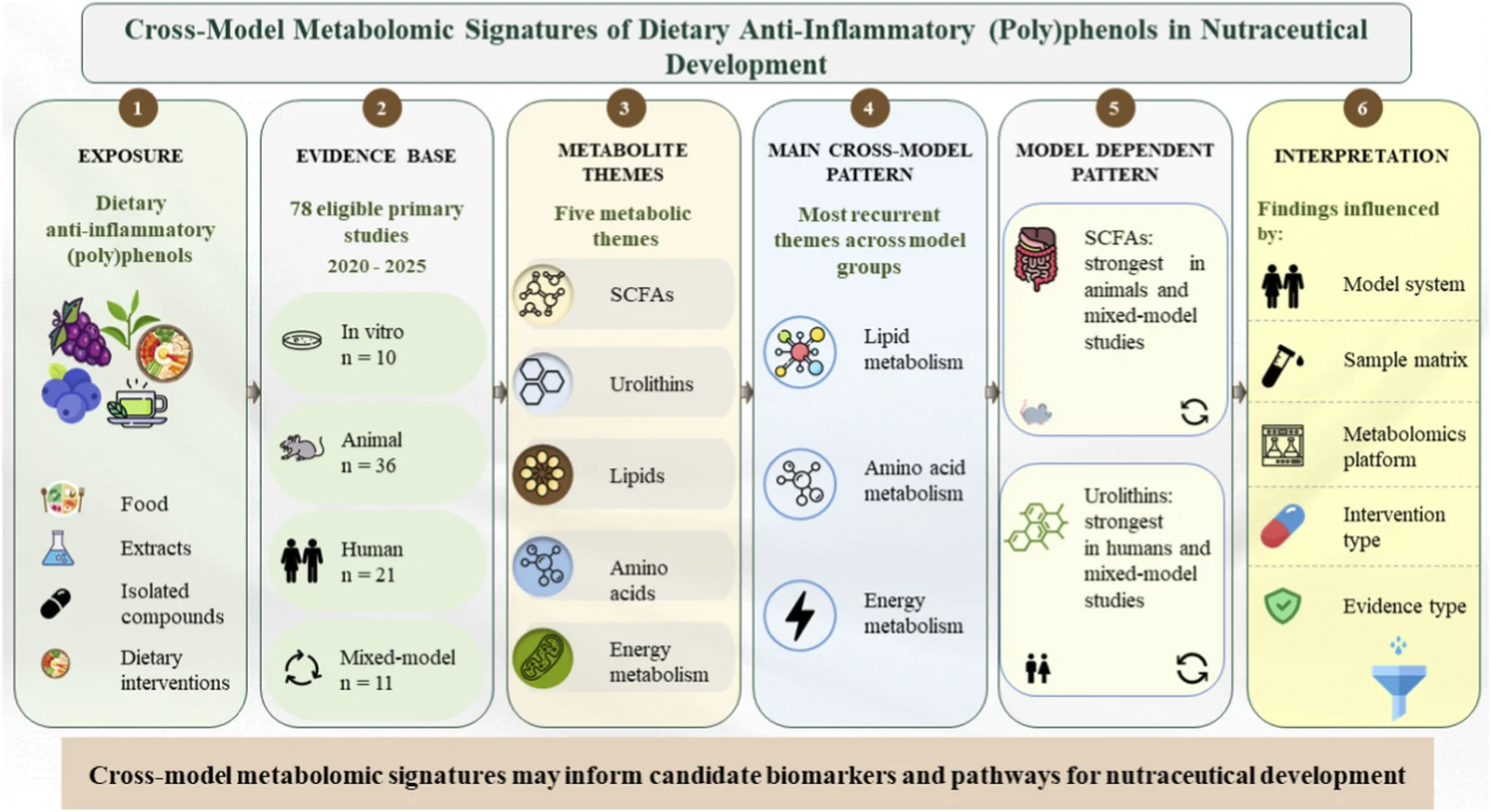
Overview of review scope, model systems, metabolite themes, recurrence patterns, and evaluation of findings across 78 eligible primary studies on dietary anti-inflammatory (poly) phenols. Graphical abstract developed by the authors. Icons were adapted from Flaticon.com.

## 1 Introduction

Dietary (poly) phenols are a diverse family of secondary metabolites of plants, characterized by one or more aromatic rings bearing hydroxyl substituents (Yaskolka Meir et al., 2021; Kasprzak-Drozd et al., 2021). They are primarily classified into flavonoids, phenolic acids, lignans, stilbenes, and tannins. Flavonoids include subclasses such as flavonols, anthocyanins, catechins, flavones, and isoflavones, while phenolic acids comprise hydroxybenzoic and hydroxycinnamic acid derivatives. Stilbenes include resveratrol-like compounds, whereas tannins and ellagitannins are larger polyphenolic structures (Yaskolka Meir et al., 2021; Hui et al., 2023; Pinto et al., 2023; Tang W. et al., 2022; Xu et al., 2025; Correia et al., 2020; Yu et al., 2025; Lackner et al., 2024). These compounds are found in commonly consumed plant-based foods, including fruits, vegetables, legumes, nuts, tea, coffee, and cocoa (Yaskolka Meir et al., 2021; Chamberlin et al., 2024; Hou et al., 2023; Sun et al., 2021; Wood et al., 2023). Interest in these (poly) phenols has grown as studies have associated their consumption with various health benefits involving inflammation (He et al., 2023; Lv et al., 2025; Tang et al., 2024; Tang L. et al., 2022), oxidative stress (Tuerson et al., 2025; Al-Aubaidy et al., 2021), metabolic regulation (Casani et al., 2024; Calabrese et al., 2024; Zhou et al., 2024; Keshteli et al., 2022; Tian et al., 2025), neuroprotection (Ávila-Gálvez et al., 2025a), and even heat stress modulation (Feng et al., 2024) (Figure 1). Several mechanisms may contribute to these responses. Dietary (poly) phenols can modulate gut microbial composition (Zhou et al., 2024; Peron et al., 2021; Abu Bakar Sajak et al., 2021), promote the production of short-chain fatty acids (SCFAs) involved in metabolic and inflammatory regulation (Keshteli et al., 2022; Abdul Kadir et al., 2020), interact with bacterial cell membranes and cellular functions (Xu et al., 2025), and influence gut immune and metabolic signaling (García-Gavilán et al., 2023).

**FIGURE 1:**
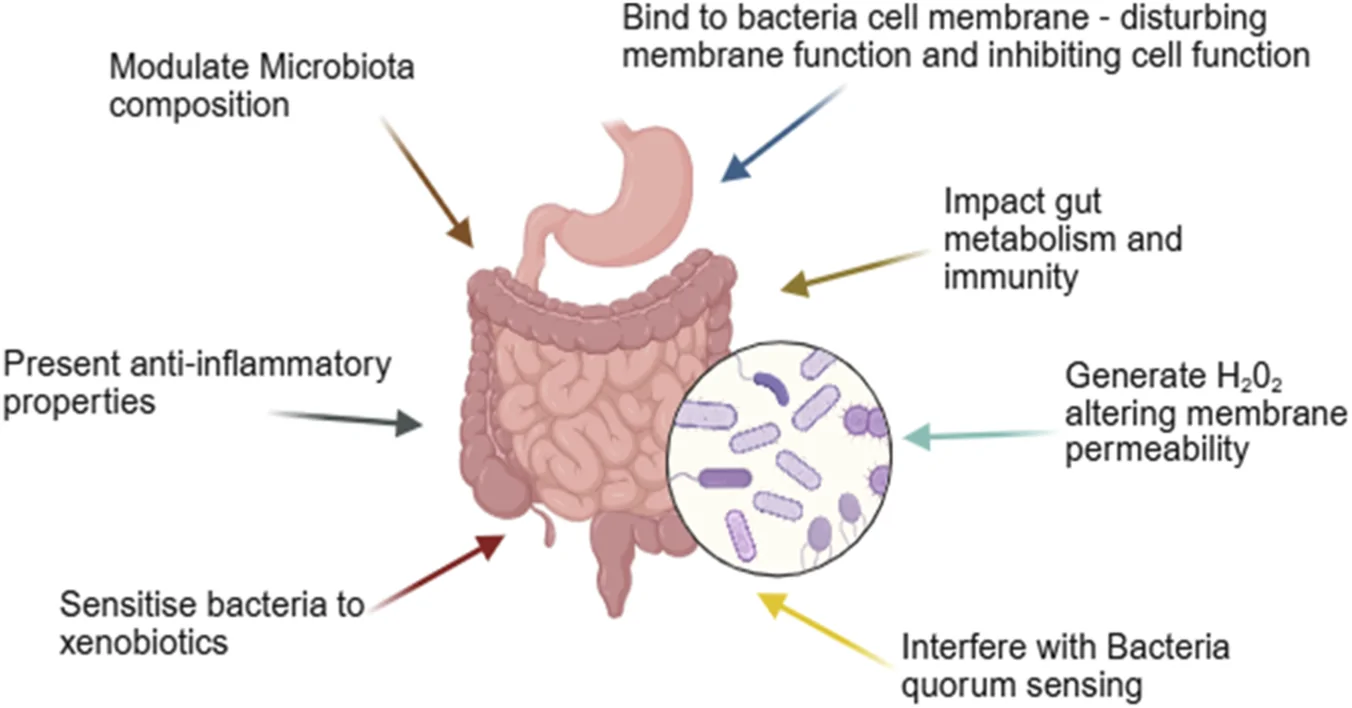
Multifunctional roles of dietary (poly) phenols in the gut. Dietary (poly) phenols may modulate gut microbiota composition, interact with bacterial cell membranes, influence gut metabolism and immunity, alter membrane permeability through hydrogen peroxide generation, interfere with bacterial quorum sensing, sensitize bacteria to xenobiotics, and exert anti-inflammatory effects. Redrawn by the authors based on concepts described by Kasprzak-Drozd et al. (Kasprzak-Drozd et al., 2021), with icons sourced from Flaticon.com. This figure is illustrative and not part of the results synthesized from the studies included.

Nutraceuticals are products derived from foods or parts of foods, including isolated nutrients, herbal products, functional foods, or modified food products, positioned at the interface of nutrition and pharmaceutical applications (Puri et al., 2022). They are generally intended to support physiological functions, promote preventive health, or reduce disease risk (Puri et al., 2022). Dietary (poly) phenols hold significant potential as nutraceuticals, given their structural diversity and documented antioxidant, anti-inflammatory, metabolic, and gut microbiota-modulating activities (Ingegneri et al., 2024; Zhang et al., 2022). Metabolomics provides a framework for elucidating the structural diversity of (poly) phenols and gaining insights into how these metabolites function, transform, and impact health. This approach extends to investigation of the gut microbiome axis, such as examining how ingested (poly) phenols are metabolized and transformed by gut microbes into smaller bioactive molecules, intermediates, and other derivative products (Chen et al., 2021; Domínguez-López et al., 2023; Zhang et al., 2023; Tsiara et al., 2025; Pomares-Bri et al., 2025). Metabolomic pathways represent interconnected biochemical routes through which molecules are synthesized, modified, degraded, transported, and exchanged within biological systems (Hou et al., 2023; Dai et al., 2024). In dietary (poly) phenol studies, this approach helps connect measured metabolites with biological functions, rather than treating each metabolite as a separate observation (Tian et al., 2025; Chen et al., 2021). The main domains associated with dietary (poly)phenols include host gut microbial co-metabolism, microbial fermentation, lipid metabolism, bile acid metabolism, amino acid metabolism, and central energy metabolism (Pinto et al., 2023; Zhou et al., 2024; Abu Bakar Sajak et al., 2021; Alaba et al., 2025). However, the results remain fragmented as most studies are examined within single models, pathways, or outcomes. This limits understanding of which metabolic pathways are consistently affected by (poly) phenols in various biological systems and which reactions are model-specific (Sun et al., 2021; Chen et al., 2021; Tsiara et al., 2025). These domains are relevant because parent (poly) phenols generally show limited small intestinal absorption, with only about 5%–10% absorbed before reaching the colon (Lackner et al., 2024; Ávila-Gálvez et al., 2025a). Most parent (poly) phenols reach the colon largely unmetabolized, where gut microbes convert them through ring cleavage, dehydroxylation, and demethylation into smaller phenolic derivatives (Pinto et al., 2023; Lackner et al., 2024; Fritsch et al., 2022a). These products may subsequently undergo host phase II conjugation, including glucuronidation, sulfation, and methylation, before systemic circulation and renal excretion (Pinto et al., 2023; Fritsch et al., 2022a; Li et al., 2022). As a result, a diverse pool of low-molecular-weight metabolites is produced (Xu et al., 2025; Hou et al., 2023; Ávila-Gálvez et al., 2025a; Messaoudene et al., 2022; Wilson et al., 2023). Importantly, the composition and degree of these metabolic responses differ substantially among individuals and experimental systems, as they are influenced by factors such as the compound class, food matrix, dose, intervention duration, host physiology, and gut microbiome. This difference in metabolic responses poses a challenge in assessing whether metabolic changes associated with (poly) phenols are reproducible in different models (Pinto et al., 2023; Tang W. et al., 2022; Xu et al., 2025; Hou et al., 2023; Messaoudene et al., 2022; Wilson et al., 2023).

In the included studies, several metabolites were investigated as indicators of dietary (poly) phenol metabolism and bioactivity. Urolithins, particularly A, B, and C, are notable microbial metabolites derived from ellagitannins, potentially connecting dietary (poly) phenol intake with metabolic responses (Tsiara et al., 2025; Yaskolka et al., 2023). SCFAs, including acetate, propionate, and butyrate, were also frequently examined as functional markers of gut microbial fermentation following (poly) phenol exposure. Amino acids were another important group, particularly branched-chain amino acids (BCAAs) and aromatic amino acids (AAAs). These metabolites may reflect metabolic stress, insulin sensitivity pathways, oxidative balance, and dietary exposure patterns (Peron et al., 2021; Bartova et al., 2022). Lipid metabolites also emerged as relevant due to their association with inflammatory lipid signaling, lipid and glucose regulation, and mitochondrial function (Crescenzi et al., 2025). Additionally, intermediates of the tricarboxylic acid (TCA) cycle and central carbon metabolism were highlighted as important indicators of shifts in energy metabolism and mitochondrial activity following (poly) phenol exposure (García-Gavilán et al., 2023; Pomares-Bri et al., 2025).

Metabolite coverage in dietary (poly) phenol research depends on the analytical platform, workflow, and biological matrix used (Clarke et al., 2023; Emwas et al., 2025). Liquid chromatography-mass spectrometry (LC-MS)-based approaches generally provide broad sensitivity for detecting bioavailable phenolic metabolites, phase II conjugates, bile acids, lipid species, amino acids, acylcarnitines, and inflammatory lipid mediators (García-Gavilán et al., 2023; Fritsch et al., 2022a; Li et al., 2022; Falcone and Rushing, 2025). Furthermore, gas chromatography-mass spectrometry (GC-MS) is particularly useful for volatile or derivatized metabolites, including SCFAs, organic acids, and other microbial fermentation products (Ávila-Gálvez et al., 2025a; Chen et al., 2021; Clarke et al., 2023). Although GC-MS is commonly used for SCFA profiling, targeted LC-MS methods can also detect SCFAs when appropriate derivatization or method optimization is applied (Saha et al., 2021; Trivedi et al., 2022). In contrast, proton nuclear magnetic resonance (^1^H-NMR) spectroscopy provides highly reproducible and non-destructive profiling of abundant low-molecular-weight metabolites, including glucose, lactate, amino acids, and organic acids, but is generally less sensitive than mass spectrometry for low abundance dietary metabolites (Lackner et al., 2024; Bartova et al., 2022; Emwas et al., 2025). Sample matrix selection also affects the metabolite coverage (Emwas et al., 2025; Chen LH. et al., 2025). Urine is useful for detecting excreted sulfated and glucuronidated conjugates, fecal and cecal samples are important for microbial metabolites and gut biotransformation products, and serum or plasma provides a systemic view of circulating metabolic changes (Tsiara et al., 2025; Fritsch et al., 2022a; Emwas et al., 2025; Chen LH. et al., 2025). While targeted methods offer more precise quantification of specific compounds, untargeted metabolomics facilitates the discovery of broad metabolic alterations. These differences can also complicate comparisons between studies, as variability and apparent inconsistencies in study findings may be attributable to differences in platform sensitivity and specificity, sample preparation, metabolite annotation confidence, and metabolite coverage (Clarke et al., 2023; Emwas et al., 2025).

Previous reviews have examined the biochemical properties of (poly) phenols, their interactions with the gut microbiota, and their effects in different disease contexts. These reviews have summarized the proposed mechanisms and biological outcomes. However, these studies stop short of asking whether these metabolomic findings are consistent in different experimental systems or if the responses observed in cell or animal models are also seen in human studies. In addition, the metabolomic results are discussed descriptively, with the metabolites or pathways highlighted within specific studies, but without a systematic attempt to assess how often the same pathways or metabolites recur in different models. As a result, despite the growing volume of metabolomics data in this field, it remains uncertain which (poly)phenol-induced metabolic changes represent stable, conserved biological responses and which are more context-dependent. To address this gap, the review brings together reports from experimental studies examining dietary anti-inflammatory (poly) phenols *in vitro* models, animal studies, human and mixed-model interventions, focusing on cross-model recurrence in metabolomic signatures, identifying pathways and metabolites that appear repeatedly in biological systems and those that remain model-specific. In doing so, the review aims to clarify how current metabolomics findings may inform nutraceutical development and guide future human studies.

## 2 Methodology

This systematic review was conducted and reported in accordance with the Preferred Reporting Items for Systematic Reviews and Meta-Analyses (PRISMA) 2020 guidelines (Page et al., 2021). A review protocol describing the objectives, eligibility criteria, and planned methods was developed and registered with the Open Science Framework (OSF) under the title “Cross-Model Metabolomic Signatures of Dietary Anti-Inflammatory (Poly) phenols in Nutraceutical Development: A Systematic Review”. The registration record is accessible at https://osf.io/5me8x/metadata/osf.

### 2.1 Information sources and search strategy

Literature searches were conducted in PubMed, Scopus, Web of Science, and Google Scholar to identify records on metabolomics, dietary polyphenols, inflammation, and nutraceutical or therapeutic uses. The Boolean search combinations included (“metabolomics” OR “metabolic profiling” OR “metabolite analysis”) AND (“polyphenol” OR “polyphenols” OR “flavonoid” OR “flavonoids” OR “phenolic compound” OR “phenolic compounds” OR “dietary polyphenol” OR “plant phenolic” OR “antioxidant polyphenol”) AND (“nutraceutical” OR “functional food” OR “dietary supplement” OR “therapeutic agent” OR “bioactive compound” OR “natural product”) AND (“anti-inflammatory” OR “inflammation” OR “oxidative stress” OR “cytokine modulation”). Additional search combinations included (“metabolomics” OR “metabolic profiling” OR “metabolite analysis”) AND (“polyphenol” OR “polyphenols” OR “flavonoid” OR “flavonoids” OR “phenolic compound” OR “phenolic compounds”) (“polyphenol” OR “polyphenols” OR “flavonoid” OR “flavonoids”) AND (“anti-inflammatory” OR “inflammation modulation” OR “oxidative stress”) (“nutraceutical” OR “functional food” OR “dietary supplement”) AND (“polyphenol” OR “polyphenols”) AND (“anti-inflammatory” OR “inflammation”) AND (“metabolomics” OR “metabolic profiling”); and (“nutraceutical” OR “functional food” OR “dietary supplement”) AND “polyphenol” AND “anti-inflammatory” AND “metabolomics”. Backward citation tracking was not performed.

### 2.2 Eligibility criteria

Studies were eligible for inclusion if they met all of the following criteria: original research involving *in vitro*, animal, human, or mixed-model designs; investigated dietary anti-inflammatory (poly) phenols or (poly)phenol-rich extracts, nutraceuticals, or dietary interventions; employed metabolomics analysis using at least one platform including GC-MS, NMR, LC-MS, LC-MS-MS, ultra-high-performance liquid chromatography-mass spectrometry (UHPLC/UPLC-MS), or high-resolution mass spectrometry (HRMS); reported metabolomic profiles, metabolite changes, or pathway-level outputs relevant to inflammation; were published in English; and were published between 2020 and 2025. Reviews, conference abstracts without full results, protocols, computational studies without biological validation, phytochemical studies without biological or metabolomic evaluation, non-(poly) phenol interventions, studies without metabolomics, studies outside the review scope, and non-English publications were excluded.

### 2.3 Study selection

Records identified from the database searches were imported into Zotero for duplicate removal. The remaining citations were transferred to Rayyan for screening. Subsequently, titles and abstracts were screened against the eligibility criteria and the potentially eligible full-text articles were thoroughly assessed for inclusion. Initially, 87 studies passed the full-text screening. However, nine were later excluded during the final verification phase for not meeting the eligibility criteria, leaving a total of 78 primary studies included in the review. The PRISMA flow diagram outlines the number of records identified, screened, excluded, and included (Figure 2).

**FIGURE 2:**
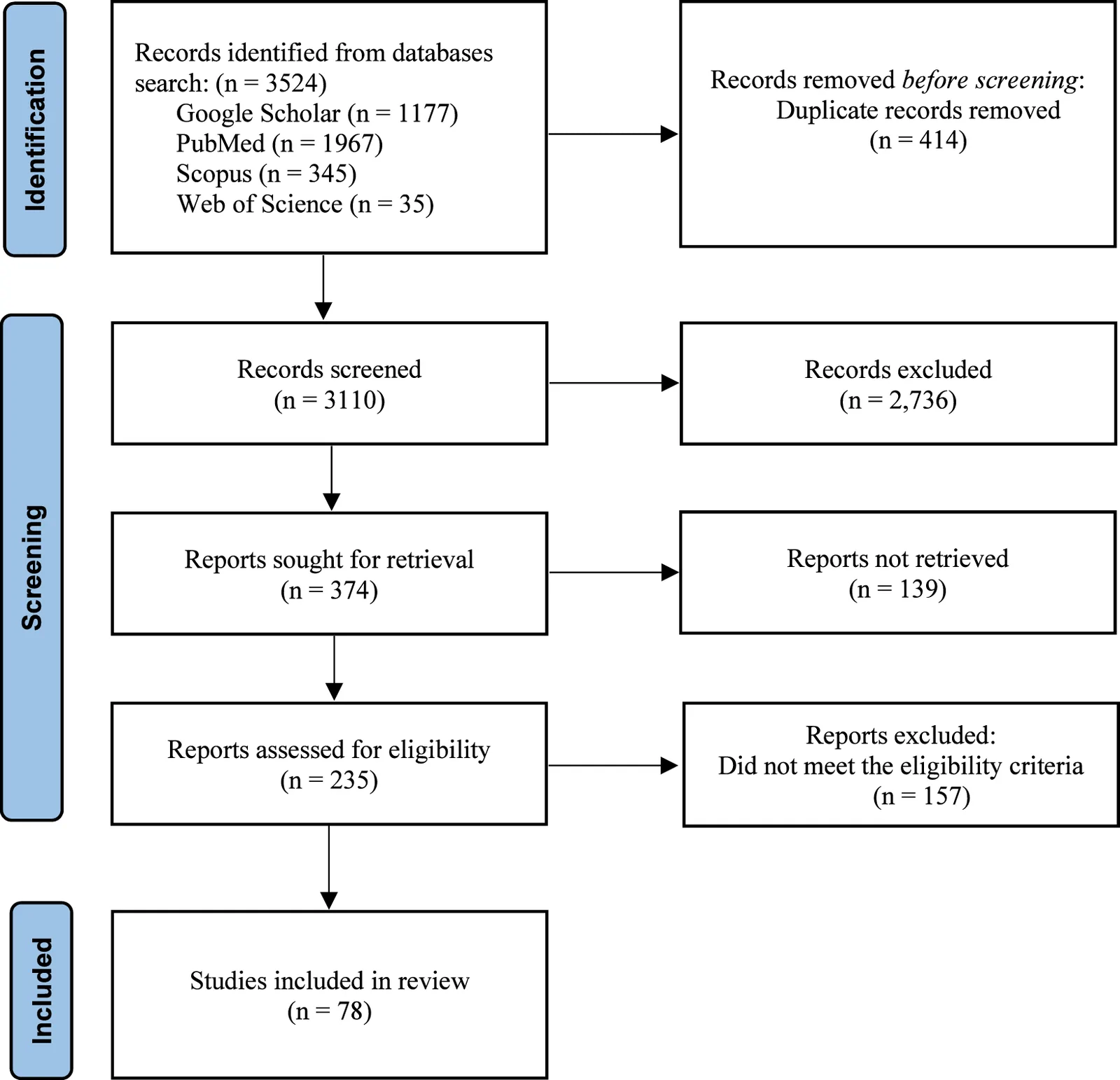
PRISMA flow diagram of information search for the systematic review (Page et al., 2021).

### 2.4 Data extraction

Microsoft Excel was used to extract variables using defined fields. These included study identification, first author, year, country, study design, model system, population or sample size, (poly) phenol intervention, intervention source, dose, duration, formulation, metabolomics platform, sample type, major biological or clinical outcomes, direction of metabolite change where reported, and risk-of-bias (RoB) assessment. The risk of bias and reporting quality were assessed according to the study type. These included SYRCLE’s risk-of-bias tool for animal studies, Cochrane RoB 2 for randomized human intervention studies, and an adapted ROBINS-I approach for non-randomized or observational human studies. A structured checklist was used for the *in vitro* and mixed-model studies.

### 2.5 Data synthesis

The extracted variables grouped in Microsoft Excel were compared to identify recurring patterns in metabolomic alterations. Synthesis emphasized descriptive assessment of the frequency, recurrence, and model-specific distribution of metabolomic changes, with attention to whether findings were substantiated by direct metabolite measurement, modeled outputs, annotation-level features, or supporting multi-omics data. Five major metabolite themes were used to organize the synthesis: SCFAs and microbial fermentation metabolites, urolithins and ellagic acid-derived metabolites, lipid metabolites, amino acids, and energy metabolism metabolites. Findings were interpreted cautiously where studies came from pathway enrichment, modeled flux, annotation-level features, or pathogen-specific systems rather than direct host metabolite measurements.

## 3 Results

### 3.1 Study selection and characteristics

Database searches identified 3,524 records. After deduplication in Zotero and Rayyan, 3,110 unique records were screened by title and abstract, and 374 full-text articles were assessed for eligibility. Seventy-eight studies were included in the review (Figure 2). The majority consisted of animal studies (n = 36), followed by human (n = 21), mixed-model (n = 11), and *in vitro* studies (n = 10) (Table 1). These examined dietary anti-inflammatory (poly) phenols in whole-food matrices, isolated components (poly)phenol-rich extracts, and broader dietary patterns. A variety of biological matrices, such as biofluids, fecal or stool samples, tissues, cell extracts, culture media, and *ex vivo* systems, were subjected to metabolomic analysis. LC-MS-based techniques, GC-MS, NMR, high-resolution MS, and multi-omics-linked processes were the primary analytical strategies (Table 1). The results were organized around five main metabolite themes, and Table 2 provides a summary of their distribution by model systems.

**TABLE 1:** Characteristics and distribution of included studies on (poly)phenols by model groups.

Model	Studies (n)	Intervention categories	Matrices	Analytical platforms	Principal metabolite themes
Animal	36	Isolated compounds, extracts, polyphenol-rich foods	Serum, plasma, feces, cecal contents, rumen fluid, urine, and tissues	LC-MS, GC-MS, NMR, LC-MS/MS, multi-omics	SCFAs, urolithins/ellagic acid-derived metabolites, lipids, amino acids, energy metabolites
Human	21	Foods, dietary patterns, polyphenol-rich interventions, clinical/dietary exposures	Urine, plasma, serum, stool, feces	LC-MS/MS, UHPLC/UPLC-MS, HRMS, NMR	Urolithins/phase II conjugates, lipids, amino acids, energy metabolites, limited SCFAs
*In vitro*	10	Isolated compounds, formulations, extracts	Cell extracts, intracellular metabolites, culture media	LC-MS, UHPLC/UPLC-MS, HRMS, NMR	Lipids, amino acids, energy metabolites, limited intracellular acetate
Mixed	11	Combined animal, human/*ex vivo*, microbiome, bacterial, and cell-based designs	Multiple matrices, including feces, serum, plasma, tissue, bacterial cells, *ex vivo* fecal systems	LC-MS, GC-MS, NMR, microbiome-linked and multi-omics	SCFAs, urolithins, lipids, amino acids, energy metabolites

LC-MS, includes LC-MS/MS, UHPLC-MS, UPLC-MS, and HRMS-based workflows where applicable; gas chromatography-mass spectrometry (GC-MS); and NMR, includes^1^ H-NMR, spectroscopy. Mixed-model studies combined two or more experimental components, including animal, human or *ex vivo*, microbiome, bacterial, or cell-based systems. The listed biological matrices, analytical platforms, and metabolite themes are representative rather than exhaustive.

**TABLE 2:** Distribution of the five metabolite themes across model groups.

Model	SCFAs	Urolithins/ ellagic acid derivatives	Lipids	Amino acids	Energy	References
*In vitro*	Limited; intracellular acetate only	Not directly detected	Recurrent cell-specific alterations across several lipid classes	Recurrent cell-specific changes involving several amino acid pathways	TCA cycle, glycolytic, acylcarnitine, and ATP-associated changes	(Pomares-Bri et al., 2025; Falcone and Rushing, 2025; D’Elia et al., 2024; Wang et al., 2020; Deng et al., 2024)
Animal	Frequently measured but directionally variable	Limited direct detection	Broadly represented across disease models and tissues	Broad but model-dependent changes	Broad and heterogeneous changes	(Pinto et al., 2023; Tang et al., 2024; Dai et al., 2024; Chen L et al., 2025; Guirro et al., 2020)
Human	Limited and matrix-dependent	Most clearly represented in urine as urolithins and phase II conjugates	Broad but heterogeneous biofluid and stool lipidomic profiles	Heterogeneous intervention and observational results	Heterogeneous plasma, urine, and stool profiles	(Lackner et al., 2024; García-Gavilán et al., 2023; Domínguez-López et al., 2023; Tsiara et al., 2025; Bartova et al., 2022; Castellano-Escuder et al., 2022)
Mixed	Direct fecal SCFA quantification frequently paired with microbiome profiling	Microbial conversion and fecal or urinary production documented	Host lipid changes reported	Host amino acid changes reported across several biological systems	Host tissue and microbial fermentation-associated changes	(Lv et al., 2025; Messaoudene et al., 2022; Ávila-Gálvez et al., 2025b; Bian et al., 2025; Lu et al., 2024)

### 3.2 Study quality and risk of bias

The risk-of-bias findings differed across study designs. Animal studies commonly had unclear risk of bias in domains relating to sequence generation, allocation concealment, random housing, and blinding because these methods were often insufficiently reported. Randomized human studies generally ranged from low risk to some concerns, mainly because of unclear allocation concealment, missing metabolomic outcome data, and limited information on whether individual metabolite analyses were specified in advance. Observational studies were mainly limited by confounding, participant loss, and exploratory metabolite selection. *In vitro* studies commonly lacked sufficient information on biological replication, randomization, blinding, and analytical quality control. Mixed-model studies combined findings from different experimental systems, but confidence in some conclusions was limited by incomplete reporting within individual components and indirect comparisons across models.

### 3.3 *In vitro* studies

The ten *in vitro* studies investigated cell-intrinsic metabolomic responses to dietary (poly) phenol interventions in various cell models. The main findings involved lipid, amino acid, and energy metabolism (Tables 2, 3). Studies on SCFAs were limited to intracellular acetate measured in isolated cell systems (Table 3). In SH-SY5Y neuroblastoma cells, ST-65 treatment was associated with lower intracellular acetate concentrations (D’Elia et al., 2024). In LPS-activated RAW 264.7 macrophages, acetate decreased under inflammatory stimulation and did not show a clear recovery trend after 3-cinnamoyltribuloside treatment (Wang et al., 2020). Urolithins were not directly identified (Table 2).

**TABLE 3:** Model-based summary of changes in metabolite profiles and patterns after dietary (poly)phenol intervention.

Model	Dietary (poly)phenols	Main metabolites/ signatures	Direction or pattern	Quantification	References
*In vitro*	Ashwagandha (*Withania somnifera*); bacopa (*Bacopa monnieri*); *Camellia nitidissima* flowers	SCFAs: Intracellular acetate	Decreased or altered in isolated cell systems	Direct intracellular metabolite measurement	(D’Elia et al., 2024; Wang et al., 2020)
Animal	Peony flower extract; *Rosa roxburghii* fruit extract	SCFAs: Acetate, propionate, butyrate, branched-chain SCFAs, valerate, caproate	Recurrent but directionally mixed; increases and selective remodeling reported	Direct metabolite measurement	(Tang et al., 2024; Fritsch et al., 2022a; Chen L et al., 2025; Fritsch et al., 2022b)
Human	Aronia berry; grape extract	SCFAs: Stool acetate, butyrate, propionate; plasma acetate	Limited; stool SCFAs largely stable; plasma acetate condition-specific	Direct measurement plus modeled flux	(Lackner et al., 2024; Bartova et al., 2022)
Mixed	Pomegranate; lemon; orange; grape; olive; berries; walnut; chestnut	SCFAs: Fecal acetate, propionate, butyrate, isobutyrate, isovalerate, valerate	Increased in direct fecal SCFA studies with microbiome profiling	Direct metabolite measurement plus microbiome profiling	(Ávila-Gálvez et al., 2025a; Ávila-Gálvez et al., 2025b; Bian et al., 2025)
*In vitro*	No data	Urolithins: None directly reported	No direct urolithin data identified	Not identified in included *in vitro* studies	None identified
Animal	Chestnut extract; pomegranate juice; berries; walnut	Urolithins: Urolithins A, B, C, D; sulfate/methyl conjugates; related phenolic acids	Direct urolithin detection limited to one core study; other studies reported related metabolites	Direct metabolite measurement	(Pinto et al., 2023; Attia et al., 2023; Xie et al., 2022)
Human	Walnut; green tea; Mankai green shake; soybeans; raspberry powder	Urolithins: Urolithin A/C; urolithin A sulfate; urolithin A/B glucuronides; other conjugates	Consistently reported in urine; stronger than SCFA changes in human studies	Direct urinary metabolite measurement	(Domínguez-López et al., 2023; Tsiara et al., 2025; Yaskolka et al., 2023; Castellano-Escuder et al., 2022)
Mixed	Mankai; green tea; walnut; pomegranate; chestnut	Urolithins: Urolithin A, isourolithin A, urolithin B; fecal urolithin A	Microbial conversion and fecal production documented	Direct metabolite measurement plus microbiome or *ex vivo* data	(Yaskolka Meir et al., 2021; Messaoudene et al., 2022; Ávila-Gálvez et al., 2025b)
*In vitro*	Lemon verbena leaves; Olive oil; grains; grapes; berries; peanuts	Lipids: Phospholipids, sphingolipids, fatty acids, acylcarnitines, lipid mediators	Cell-specific remodeling in stress/inflammatory models	Direct metabolite measurement	(Pomares-Bri et al., 2025; Crescenzi et al., 2025; Falcone and Rushing, 2025)
Animal	Peony flower extract; kiwifruit leaves; grapes; berries; tea; walnut	Lipids: Phospholipids, sphingolipids, acylcarnitines, lipid mediators, ketone bodies	Broad but subclass-specific remodeling	Mostly direct metabolite measurement; some pathway-level data	(Hou et al., 2023; Sun et al., 2021; Chen L et al., 2025; Chao et al., 2020)
Human	Aronia juice; olive oil; Navelina oranges; cocoa; tea; apple	Lipids: Phospholipids, sphingolipids, bile acids, acylcarnitines, fatty acids, ketone bodies	Broad but heterogeneous lipidomic signatures	Direct lipidomics; observational and intervention data	(Chamberlin et al., 2024; Calabrese et al., 2024; Keshteli et al., 2022; Peron et al., 2021; García-Gavilán et al., 2023)
Mixed	*Abelmoschus manihot* flowers; *Forsythia suspensa* fruits; camu berries	Lipids: Phospholipids, bile acids, lipid mediators, ketone bodies, SCFA overlap	Host lipid patterns; pathway-level and pathogen-specific findings treated cautiously	Direct measurement plus pathway-level data	(Lv et al., 2025; Messaoudene et al., 2022; Chao et al., 2022)
*In vitro*	Lemon verbena; olive oil; grains; *Hibiscus sabdariffa*; bacopa; turmeric	Amino acids: BCAAs, AAAs, kynurenine, glutamine/glutamate, glycine/serine/threonine	Recurrent cell-specific remodeling	Direct metabolite measurement	(Pomares-Bri et al., 2025; D’Elia et al., 2024; Deng et al., 2024)
Animal	Citrus fruits; *Rosa roxburghii* fruit extracts; peony flower extracts; tea; walnut; pomegranate	Amino acids: BCAAs, AAAs, indole metabolites, kynurenic/xanthurenic acids	Broad but model-dependent changes	Direct metabolite measurement	(Sun et al., 2021; Tang et al., 2024; Chen L et al., 2025; Guirro et al., 2020; Wu et al., 2022)
Human	Aronia berry; grape extract; cocoa; tea; apple	Amino acids: BCAAs, AAAs/kynurenine metabolites, glutamine/asparagine	Heterogeneous intervention and observational findings	Direct measurement; observational data	(Chamberlin et al., 2024; Peron et al., 2021; Bartova et al., 2022; Kimble et al., 2022)
Mixed	*Abelmoschus manihot* flowers; grape; turmeric; *Forsythia suspensa* fruits; peanuts	Amino acids: BCAAs, tryptophan/5-HT, glutamine/alanine, aspartate	Host amino acid patterns; pathogen-only findings separated	Direct host measurement plus pathway-level responses	(Lv et al., 2025; Bian et al., 2025; Liu X et al., 2024; Chao et al., 2022)
*In vitro*	Olive oil; grains; *Hibiscus sabdariffa*; berries; peanut; grapes	Energy: TCA cycle intermediates, glycolysis metabolites, acylcarnitines, ATP biomarkers	Cell-intrinsic energy remodeling	Direct metabolite measurement	(Pomares-Bri et al., 2025; Falcone and Rushing, 2025; Wang et al., 2020)
Animal	Grape; tea; berries; walnuts; blueberries; *Rosa roxburghii* fruits	Energy: TCA cycle intermediates, ketone bodies, carnitines/acylcarnitines, fermentation metabolites	Recurrent but model-specific	Direct measurement plus supportive pathway or proteomic results	(Sun et al., 2021; Dai et al., 2024; Wu et al., 2022; Wang et al., 2022)
Human	Ginger; grape extract; berries; cocoa; tea; apple extract	Energy: Citrate, glucose, lactate, pyruvate, ketone bodies, acylcarnitines, ADP, adenosine, acetate	Broad but heterogeneous plasma/urine/stool signatures	Direct measurement; observational/modeled support	(Casani et al., 2024; Keshteli et al., 2022; Peron et al., 2021; García-Gavilán et al., 2023; Bartova et al., 2022)
Mixed	*Abelmoschus manihot* flowers; grapes; berries; turmeric; peanuts	Energy: Fumarate, succinate, glucose, lactate, 3-hydroxybutyrate, ADP, SCFAs	Host renal, tumor, hepatic, and microbial fermentation patterns	Direct host measurement, microbial fermentation, and pathway-level indicators	(Lv et al., 2025; Messaoudene et al., 2022; Bian et al., 2025; Liu X et al., 2024)

Several studies reported changes in lipid classes, including phospholipids, sphingolipids, fatty acids, acylcarnitines, lipid mediators, and selected bile acids or ketone bodies (Table 3). In hyperglycemic Simpson-Golabi-Behmel syndrome (SGBS) adipocytes, verbascoside and ferulic acid countered reductions in multiple phospholipid and lysophospholipid classes, including LPC, PC, PE, PI, and PS species (Pomares-Bri et al., 2025). Phospholipid metabolism also differed in heat-stressed intestinal epithelial cells treated with mung bean (poly) phenols and LPS-activated macrophages treated with 3-cinnamoyltribuloside. In the macrophage study, choline was measured as a phospholipid precursor rather than a specific phospholipid species (Feng et al., 2024; Wang et al., 2020). Sphingolipid and fatty acid alterations were identified in BEAS-2B bronchial epithelial cells, where fennel-derived metabolites altered ceramide, ceramide-1-phosphate, and polyunsaturated fatty acid profiles (Crescenzi et al., 2025). Acylcarnitines were identified in resveratrol-treated breast cancer cells and curcumin-treated chondrocytes (Falcone and Rushing, 2025; Deng et al., 2024). Curcumin-treated chondrocytes also showed altered prostaglandin mediators (Deng et al., 2024).

In hyperglycemic SGBS adipocytes, verbascoside and ferulic acid countered model-associated reductions in BCAAs, AAAs, and metabolites linked to glutamate, glutamine, arginine, proline, glycine, serine, and threonine metabolism (Pomares-Bri et al., 2025). Curcumin-treated chondrocytes showed changes involving hydroxykynurenine, glutamine, arginine, proline, and glycine/serine/threonine pathways (Deng et al., 2024). In LPS-activated RAW 264.7 macrophages, 3-cinnamoyltribuloside altered the levels of glycine, alanine, phenylalanine, pyroglutamate, and glutamate metabolites. Additional findings were reported in SH-SY5Y neuroblastoma cells treated with ST-65, where leucine, valine, tryptophan, proline, glutamate, tyrosine, phenylalanine, and serine levels were altered. Mung bean polyphenol-treated intestinal epithelial cells also showed changes in dipeptide markers, including phenylalanine-glycine (Feng et al., 2024; D’Elia et al., 2024).

The findings pertaining to energy metabolism encompassed TCA cycle intermediates, glycolysis-associated metabolites, acylcarnitines, ATP biomarkers, and intracellular acetate (Table 3). In hyperglycemic SGBS adipocytes, verbascoside and ferulic acid countered model-associated decreases in TCA cycle intermediates, including citrate, malate, succinate, aconitate, and 2-oxoglutarate, as well as glycolysis metabolites such as glucose, pyruvate, lactate, glycerol-3-phosphate, and phosphoenolpyruvate (Pomares-Bri et al., 2025). Curcumin-treated chondrocytes showed changes in malic acid, pyruvate, 2-phosphoglyceric acid, phosphoenolpyruvate, and palmitoylcarnitine, while 3-cinnamoyltribuloside altered glucose responses in LPS-activated macrophages (Wang et al., 2020; Deng et al., 2024). Acylcarnitine was identified in resveratrol-treated breast cancer cells, curcumin-treated chondrocytes, and mung bean polyphenol-treated intestinal epithelial cells (Feng et al., 2024; Falcone and Rushing, 2025; Deng et al., 2024). Mitochondrial mass was measured alongside energy metabolism outcomes in a study (Pomares-Bri et al., 2025). Overall, these are consistent with intrinsic cell alterations in lipid, amino acid, and energy metabolism. Acetate was detected only as an intracellular SCFA, and no *in vitro* study directly measured urolithin (Figure 3).

**FIGURE 3:**
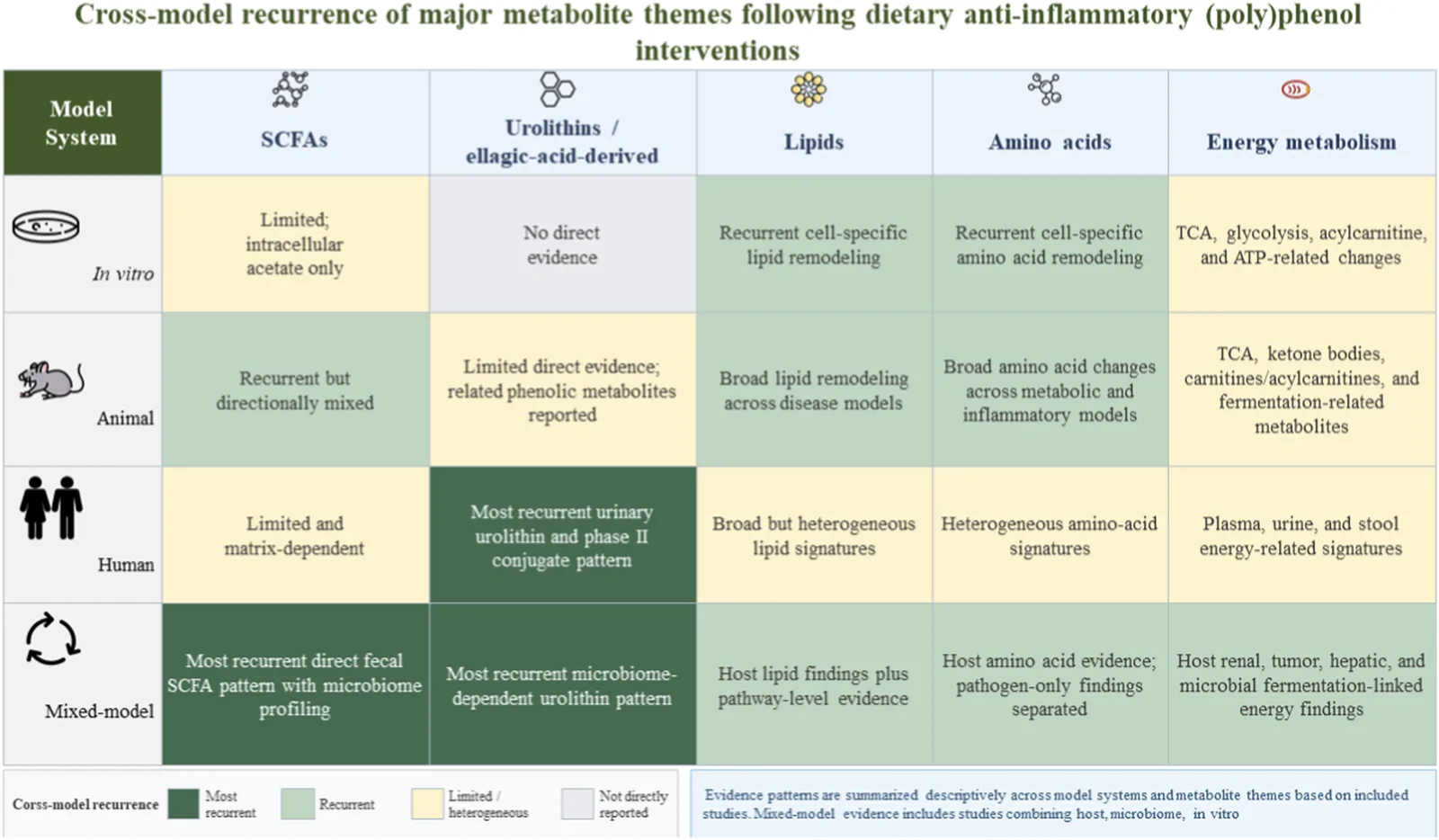
Cross-model recurrence of major metabolite themes following dietary (poly) phenol interventions. The figure summarizes the relative recurrence of five metabolite themes *in vitro*, animal, human, and mixed-model studies. Lipid metabolism, amino acid metabolism, and energy metabolism were broadly represented in model groups, while SCFAs and urolithins showed stronger model dependence. Figure developed by the authors; icons adapted from Flaticon.com.

### 3.4 Animal studies

Animal studies drew on fecal, cecal, rumen, serum/plasma, urine, tissue, and organ-specific samples from metabolic, inflammatory, cardiopulmonary, gastrointestinal, neurological, livestock, and injury models (Tables 2, 3). SCFA metabolites were frequently measured, especially in feces, cecum, rumen, serum, and tissues (Table 3). Several studies reported increases in acetate, propionate, butyrate, or total SCFAs. Peony flower polyphenols in T2DM mice, *Rosa roxburghii* polyphenols in LPS-induced acute lung injury mice, and gallic acid in stressed puppies were associated with increased SCFA concentrations (Tang et al., 2024; Yang et al., 2021; Chen L. et al., 2025). However, the direction of the SCFA response was not uniform. In canine models, dietary interventions increased selected SCFAs such as acetate or butyrate, while decreasing propionate and branched-chain SCFAs (Fritsch et al., 2022a; Fritsch et al., 2022b). Other animal studies reported reduced propionic acid or unclear directional effects, including in pomegranate-treated rats and PD-MCI mice (Tuerson et al., 2025; Attia et al., 2023).

Direct detection of urolithins was limited (Table 3). One study detected urolithin after chestnut shell extract administration in male Wistar rats (Pinto et al., 2023). Other studies reported the presence of ellagic acid or ellagitannin-derived phenolic metabolites. Ellagic acid treatment in male Sprague-Dawley rats was associated with changes in 2-hydroxyphenylacetic acid in brain tissue (Xie et al., 2022). Pomegranate juice intervention in Sprague-Dawley rats was associated with fecal microbial phenolic acids, including 3-hydroxyphenylpropionic acid and 4-hydroxyhydrocinnamic acid (Attia et al., 2023).

The lipid metabolites included phospholipids, sphingolipids, fatty acids, acylcarnitines, lipid mediators, and ketone bodies (Table 3). In metabolic disease models, peony flower polyphenols and *Actinidia arguta* leaf interventions were associated with changes in serum phospholipid and lysophosphatidylcholine amounts in T2DM mice (Hou et al., 2023; Chen LH. et al., 2025). Canine and humanized mouse models also reported shifts in phosphatidylcholine after fiber/polyphenol or Aronia juice interventions (Wilson et al., 2023; Fritsch et al., 2022b). Sphingolipids and acylcarnitines appeared in inflammatory and cardiopulmonary models. *Rosa roxburghii* polyphenols altered sphinganine and phytosphingosine in an LPS-induced acute lung injury model, while resveratrol treatment was associated with 2-hexenoylcarnitine changes in pulmonary arterial hypertension rats (Sun et al., 2021; Tang et al., 2024). Livestock and companion animal models showed changes in lipid mediators after (poly) phenol ingestion (Yu et al., 2025; Yang et al., 2025). Colitis and nonalcoholic fatty liver disease (NAFLD)/diabetes models showed alterations in ketone bodies (Zhou et al., 2024; Chao et al., 2020).

Amino acids appeared in metabolic, inflammatory, gastrointestinal, cardiopulmonary, livestock, and neurological models (Table 3). Metabolic disease models treated with hesperidin, resveratrol, and peony flower (poly) phenols were associated with changes in BCAAs, AAAs, and related metabolites (Sun et al., 2021; Chen LH. et al., 2025; Guirro et al., 2020; Liu et al., 2023; Jeong et al., 2021). In acute inflammation and gastrointestinal models, gallic acid, *Rosa roxburghii* polyphenols, and fiber/polyphenol interventions were associated with changes in isoleucine, indole-3-acetic acid, 5-HIAA, indole-3-lactic acid, indoleacetate, indolepropionate, leucine, and tryptophan-derived metabolites (Tang et al., 2024; Fritsch et al., 2022b; Wu et al., 2022). Additional urinary metabolites from tryptophan catabolism were reported in APCmin/+ mice, where honokiol altered kynurenic acid and xanthurenic acid (Chen et al., 2021).

Energy metabolism involved glycolysis metabolites, ketone bodies, carnitines/acylcarnitines, mitochondrial markers, and microbial fermentation metabolites (Table 3) (Belcher et al., 2024). Changes in TCA cycle intermediates were also reported in several models. These included tissue-specific increases in citric, succinic, and malic acids after stilbene administration; increased citric acid or fumarate after resveratrol treatment in pulmonary arterial hypertension (PAH) rats; and changes in malic or succinic acid after gallic acid treatment in acute inflammation models (Sun et al., 2021; Dai et al., 2024; Wu et al., 2022). Succinic acid was an exception among the reported biomarkers, as it did not follow the same trend toward control levels after gallic acid intervention (Wu et al., 2022). Gallic acid increased hepatic ketone bodies in NAFLD/diabetes mice, while *Rosa roxburghii* polyphenol treatment was associated with changes in L-carnitine in diabetic mice (Chao et al., 2020; Wang et al., 2022).

Overall, the studies showed broad but uneven patterns of results. Urolithins were directly detected in only a small number of studies. Lipid, amino acid, and energy metabolism responses were more widely distributed in disease models, sample matrices, and analytical platforms, whereas SCFA responses recurred but varied in direction (Figure 3).

### 3.5 Human studies

The studies analyzed urine, plasma, serum, stool, or fecal samples and included dietary interventions, clinical cohorts, observational analyses, and risk-stratified populations (Table 3). In the Aronia juice intervention, stool acetate, butyrate, and propionate were directly measured but remained largely stable, with no significant shift in SCFA concentration after Bonferroni correction. In the same study, modeled microbial flow analysis, rather than direct SCFA quantification, indicated higher acetate production in the tolerance group (Lackner et al., 2024). In an overfeeding model, plasma acetate was measured by ^1^H-NMR and differed between placebo and grape polyphenol conditions (Bartova et al., 2022).

Urolithin results were obtained mainly from urinary metabolomics (Table 3). In the Mediterranean and Green-MED dietary intervention study, urinary urolithin A and urolithin C were elevated in groups that included walnut intake, alongside biological age attenuation outcomes (Yaskolka et al., 2023). Increased urinary urolithins were reported after a short-term intervention involving polyphenol-rich foods, including raspberry powder (Tsiara et al., 2025). Observational studies identified urinary urolithins as candidate biomarkers linked to diet. Positive associations between urolithin conjugates and higher AHEI-2010 diet quality scores were reported (Castellano-Escuder et al., 2022), while higher urolithin A glucuronide, urolithin C, and urolithin B diglucuronide were reported among participants free of T2D compared with those with T2D (Domínguez-López et al., 2023; Castellano-Escuder et al., 2022). Urolithins and ellagic acid-derived metabolites were detected and annotated in plasma and urine reference materials (Li et al., 2022).

Lipid signatures included phospholipids, sphingolipids, bile acids, acylcarnitines, fatty acids, and ketone bodies (Table 3). Fecal lipid changes were reported after Aronia consumption in an intervention study, with upregulated phosphatidylserine, phosphatidic acid, phosphatidylglycerol, fecal chenodeoxycholic acid, and downregulated serum 6-octenoylcarnitine (Chamberlin et al., 2024). In the PREDIMED cohort, olive oil intake was associated with plasma lipidomic profiles, including plasmalogens, lysophosphatidylethanolamines, sphingomyelins, ceramides, bile acids, and acylcarnitines. The derived metabolite profiles were inversely associated with cardiovascular disease incidence and were recorded as diet-associated lipid signatures (García-Gavilán et al., 2023). Additional studies documented phosphatidylcholine profiles in ulcerative colitis (UC) patients receiving an anti-inflammatory diet, reductions in selected serum acylcarnitines after a polyphenol-rich diet in older adults, and fatty acid/VOC signatures in MAFLD patients (Calabrese et al., 2024; Keshteli et al., 2022; Peron et al., 2021).

Metabolomic alterations linked to amino acids were also observed (Table 3). The most distinct BCAA pattern came from an overfeeding study in which grape polyphenol supplementation attenuated increases in circulating BCAA levels (Bartova et al., 2022). Other intervention studies reported changes in individual amino acids, including increased serine and phenylalanine after multi-component supplementation, increased asparagine and tyrosine after Aronia juice consumption, and decreased serum asparagine after a polyphenol-rich diet in older adults (Chamberlin et al., 2024; Peron et al., 2021; Kimble et al., 2022). The findings involving kynurenine and AAAs were more heterogeneous. These included urinary kynurenine following a polyphenol-rich dietary intervention, subset-specific reductions in indoxyl sulfate, and observational associations between serotonin metabolites or microbial AAA catabolites and cognitive decline (Correia et al., 2020; Domínguez-López et al., 2023; Lin et al., 2023). Changes in urine anserine, lysine, and urea following supplementation, variations in urinary p-hydroxybenzoic acid following an anti-inflammatory diet, and variations in glutamine by Aronia tolerance group were further reported (Lackner et al., 2024; Casani et al., 2024; Keshteli et al., 2022).

TCA cycle intermediates, glycolysis metabolites, ketone bodies, acylcarnitines, alterations in metabolites linked to ATP and microbial fermentation were among the findings in energy metabolism studies (Table 3). In the overfeeding study, grape polyphenol supplementation was associated with differences in plasma citrate, glucose, lactate, pyruvate, ketone bodies, and acetate compared with the overfeeding/placebo condition (Bartova et al., 2022). In a large cohort study, olive oil intake was associated with plasma citrate, malate, lactate, fructose-6-phosphate, acylcarnitines, adenosine diphosphate (ADP), and adenosine. These were treated as diet-associated metabolomic signatures rather than intervention-specific responses (García-Gavilán et al., 2023). Additional clinical studies reported succinate, fumarate, pyruvate, acetone, methyl succinate, and carnitine-linked metabolites in the contexts of UC, benign paroxysmal positional vertigo (BPPV), aging, and diet quality. Findings involving microbial energy metabolism included stable stool SCFA concentrations with modeled microbial flux outputs after Aronia intake, as well as plasma acetate responses during overfeeding (Lackner et al., 2024; Bartova et al., 2022).

For urinary urolithins and phase II conjugates, human studies generally produced the strongest results, while SCFA results were more restricted and sample matrix-dependent (Figure 3). The findings on lipid metabolism, amino acid metabolism, and energy metabolism were extensive yet diverse. This included directly measured metabolites from intervention studies, as well as observational, modeled, or annotation-level results.

### 3.6 Mixed-model

Studies that combined fecal metabolite measurement with gut microbial profiling gave the most definitive results for acetate, propionate, butyrate, isobutyrate, isovalerate, valerate, and total SCFAs (Table 3). Encapsulated ellagic acid increased these metabolites in male BALB/c mice, with fecal microbial profiling conducted alongside metabolite measurement (Ávila-Gálvez et al., 2025a; Ávila-Gálvez et al., 2025b). In the kennel dog resveratrol study, fecal SCFAs were directly quantified, with butyrate and isovalerate increasing over time in the resveratrol group. The study also included 16S rRNA sequencing data (Bian et al., 2025). Ellagitannin and ellagic acid interventions yielded measurable urolithin metabolites in several systems (Table 3). In *ex vivo* fecal systems, castalagin was converted by human fecal microbiota into urolithins in samples from most clinical responders (Messaoudene et al., 2022). In the Green Mediterranean diet study, urinary urolithin A increased in the intervention group (Yaskolka Meir et al., 2021). Encapsulated ellagic acid reportedly increased fecal urolithin A production in mice in a sex-dependent manner, with the highest concentrations reported in males (Ávila-Gálvez et al., 2025b). However, urolithins were reportedly not detected in plasma within the 24-h sampling window (Ávila-Gálvez et al., 2025b).

Lipid profiles covered host phospholipids, bile acids, lipid mediators, ketone bodies, and metabolites overlapping with microbial fermentation (Table 3). In host tissues, phospholipid changes included increased hepatic phospholipids and altered phosphatidylethanolamine patterns in high-fat diet and metabolic dysfunction-associated fatty liver disease models, along with changes in sn-glycero-3-phosphocholine in acute kidney injury kidney extracts (Lv et al., 2025; Liu X. et al., 2024). Bile acids appeared after castalagin and ellagic acid exposure, including increased taurine-conjugated bile acids in tumor and serum samples and increased plasma cholic acid after ellagic acid treatment (Messaoudene et al., 2022; Ávila-Gálvez et al., 2025b). In a dextran sulfate sodium colitis model, *Forsythia suspensa* extract reduced model-associated increases in oxidized linoleic acid derivatives, including 13-L-hydroperoxylinoleic acid (Chao et al., 2022).

Changes in amino acids were reported in host tissues, animal biofluids, and pathogen-specific systems (Table 3). In DSS-colitis mice, *Forsythia suspensa* extract reversed model-associated increases in serum leucine, isoleucine, and aspartic acid, while also reversing reduced serum tryptophan levels (Chao et al., 2022). In folic acid-induced (FA-induced) acute kidney injury (AKI) mice, curcumin altered multiple kidney amino acid levels, including BCAAs, tryptophan/5-HT metabolites, alanine, glutamine, proline, creatine, and threonine; some metabolites increased further rather than moving toward control (Liu X. et al., 2024). Changes in tryptophan, serotonin, and related neuroactive metabolites were observed in serum, feces, brain, and colon samples in resveratrol-treated kennel dogs and mouse validation models (Bian et al., 2025). In MAFLD models, additional hepatic findings involved serine, threonine, arginine, and glutamate pathways.

Energy metabolism was reflected through host tissue metabolites, tumor-specific metabolic shifts, nucleotide-associated metabolites, and substrates linked to microbial fermentation (Table 3). In folic acid-induced AKI mice, curcumin altered renal fumarate, succinate, glucose, lactate, and 3-hydroxybutyrate, as measured by NMR. Some metabolites deviated further from control values rather than normalizing (Liu X. et al., 2024). In castalagin-treated sarcoma mice, malic acid was reduced in tumor tissue. In hepatic MAFLD models, treatment was associated with pathway-level changes in central carbon metabolism; these were used to contextualize the metabolic response rather than as definitive metabolite level results (Lv et al., 2025; Messaoudene et al., 2022). In dextran sulfate sodium (DSS)-induced colitis mice, the flavonoid extract of *Polygonum viviparum* L (TFPV) reduced fecal adenosine diphosphate (ADP) and inosine, and this reduction was discussed in relation to purine metabolism and lower uric acid levels (Liu et al., 2025). Findings involving microbial fermentation overlapped with the SCFA theme. These included increased fecal butyric acid and isovaleric acid in resveratrol-treated dogs, as well as increased fecal acetate, propionate, and butyrate after encapsulated ellagic acid treatment in male mice (Ávila-Gálvez et al., 2025b; Bian et al., 2025).

Overall, the most distinct metabolomic patterns observed in this model were comprised of fecal SCFAs, urolithins, certain host lipid and amino acid metabolites, as well as energy markers from renal or microbial fermentation (Figure 3).

### 3.7 Cross-model

The comparison showed clear differences in how the five metabolite themes appeared in the different studies (Table 4). Changes in SCFAs recurred mainly in animal and mixed-model studies (Tang L. et al., 2022; Fritsch et al., 2022a; Yang et al., 2021; Chen L. et al., 2025; Fritsch et al., 2022b). Mixed-model studies added clearer gut-focused measurements when fecal SCFA quantification was combined with microbial profiling (Ávila-Gálvez et al., 2025b; Bian et al., 2025). Human studies showed a more limited and matrix-dependent pattern (Lackner et al., 2024; Bartova et al., 2022). *In vitro* studies were more restricted, with acetate reported only as an intracellular metabolite (D’Elia et al., 2024; Wang et al., 2020). Human studies reported urinary urolithins and phase II conjugates (Domínguez-López et al., 2023; Tsiara et al., 2025; Yaskolka et al., 2023; Castellano-Escuder et al., 2022), while mixed-model studies further reported microbiome-dependent urolithin production from castalagin or ellagic acid interventions (Messaoudene et al., 2022; Ávila-Gálvez et al., 2025b). Direct urolithin detection in animal-only studies was limited, with most of the results stemming from one study on chestnut shell extract. No *in vitro* study directly detected urolithins. Lipid metabolites recurred in all four model groups but differed by lipid subclass and sample matrix (Table 4). *In vitro* studies reported cell-specific changes in phospholipids, sphingolipids, fatty acids, acylcarnitines, and lipid mediators (Pomares-Bri et al., 2025; Crescenzi et al., 2025; Falcone and Rushing, 2025; Bian et al., 2025). Animal studies reported broader lipid variation in metabolic, inflammatory, cardiopulmonary, livestock, and gastrointestinal models (Hou et al., 2023; Sun et al., 2021; Tang L. et al., 2022; Chen L. et al., 2025; Chao et al., 2020). Human studies documented heterogeneous lipidomic signatures in both intervention and cohort designs (Chamberlin et al., 2024; Calabrese et al., 2024; Keshteli et al., 2022; Peron et al., 2021; García-Gavilán et al., 2023). Studies combining multiple models captured host lipid changes as well, while findings associated with SCFAs were discussed under microbial fermentation and pathogen-specific lipid changes were kept separate (Lv et al., 2025; Messaoudene et al., 2022; Liu X. et al., 2024; Chao et al., 2022). Amino acid metabolism was also represented in the model groups, but its direction and interpretation varied by model and matrix (Table 4). *In vitro* models showed cell-level amino acid pathways (Feng et al., 2024; Pomares-Bri et al., 2025; D’Elia et al., 2024; Wang et al., 2020; Deng et al., 2024). Animal and human studies extended this theme to metabolic, inflammatory, gastrointestinal, cardiopulmonary, and neurological models (Sun et al., 2021; Lv et al., 2025; Chen et al., 2021; Chen L. et al., 2025; Fritsch et al., 2022b; Guirro et al., 2020; Kimble et al., 2022; González‐Domínguez et al., 2021). In mixed-model studies, host-derived amino acid changes were reported in colitis, AKI, metabolic dysfunction-associated fatty liver disease, and stress-related models (Xu et al., 2025; Lv et al., 2025; Bian et al., 2025; Liu X. et al., 2024; Gao et al., 2022). Patterns in energy metabolism appeared in all four model groups, although the metabolites and biological contexts differed (Table 4). *In vitro* studies mainly captured cell-intrinsic shifts in central carbon metabolism and acylcarnitines (Feng et al., 2024; Pomares-Bri et al., 2025; Falcone and Rushing, 2025; Wang et al., 2020; Deng et al., 2024). Animal studies reported a wider range of energy metabolism changes in disease models (Sun et al., 2021; Dai et al., 2024; Fritsch et al., 2022a; Chen L. et al., 2025; Chao et al., 2022). Human studies showed biofluid- and stool-based energy metabolism findings (Keshteli et al., 2022; García-Gavilán et al., 2023; Bartova et al., 2022; Castellano-Escuder et al., 2022; González‐Domínguez et al., 2021). In mixed-model studies, energy metabolism was represented through host renal, tumor, hepatic, nucleotide-linked, and microbial fermentation-linked metabolites (Figure 3) (Xu et al., 2025; Lv et al., 2025; Messaoudene et al., 2022; Bian et al., 2025; Liu X. et al., 2024; Liu et al., 2025; Gao et al., 2022).

**TABLE 4:** Cross-model recurrence and divergence of metabolomic signatures.

Metabolites	Models					References
	*In vitro*	Animal	Human	Mixed	Cross	
SCFAs	Limited to intracellular acetate; not microbial production	Recurrent but directionally mixed; fecal, cecal, rumen, serum, and tissue-based changes reported	Limited and matrix-dependent; stool SCFAs were largely stable in one study, while plasma acetate showed condition-specific changes in another	Most directly captured in studies where direct fecal SCFA quantification was paired with gut microbial profiling	Strong recurrence in animal and mixed gut studies; weak *in vitro* and limited in humans	(Lackner et al., 2024; Fritsch et al., 2022a; Bartova et al., 2022; D’Elia et al., 2024; Wang et al., 2020; Yang et al., 2021; Chen L. et al., 2025; Fritsch et al., 2022b)
Urolithins/ellagic acid-derived	No direct urolithin response identified	Limited direct detection; one core study measured urolithins A to D and conjugates, while other studies detected related phenolic metabolites	Strong urinary pattern for urolithins and phase II conjugates as exposure or response biomarkers	Strong pattern for microbial conversion and fecal or urinary urolithin production	Most recurrent in human and mixed-model studies; limited in animal studies; not directly reported *in vitro* studies	(Pinto et al., 2023; Domínguez-López et al., 2023; Tsiara et al., 2025; Messaoudene et al., 2022; Yaskolka et al., 2023; Attia et al., 2023; Castellano-Escuder et al., 2022)
Lipid	Recurrent cell-specific changes in phospholipids, sphingolipids, fatty acids, acylcarnitines, and lipid mediators	Broad changes in lipid metabolism in metabolic, inflammatory, cardiopulmonary, livestock, and gastrointestinal models	Broad but heterogeneous lipidomic signatures in intervention and cohort studies	Host lipid changes; pathway-level results and overlap with SCFA patterns interpreted independently; pathogen-specific findings separated	Recurrent in all model groups, but lipid subclass and sample matrix varied substantially	(Chamberlin et al., 2024; Hou et al., 2023; Sun et al., 2021; García-Gavilán et al., 2023; Pomares-Bri et al., 2025; Crescenzi et al., 2025; Falcone and Rushing, 2025; D’Elia et al., 2024; Deng et al., 2024; Liu X. et al., 2024; Chao et al., 2022)
Amino acids	Recurrent cell-specific changes in BCAAs, AAAs/kynurenine, glutamate/glutamine, arginine/proline, and glycine/serine/threonine pathways	Broad patterns in metabolic, inflammatory, gastrointestinal, cardiopulmonary, livestock, and neurological models	Heterogeneous patterns involving BCAAs, AAAs/kynurenine, glutamine/asparagine, and related metabolites	Host amino acid patterns reported; pathogen-only amino acid pathway results separated from host data	Recurrent in all model groups, but direction and evaluation varied by model and matrix	(Sun et al., 2021; Pomares-Bri et al., 2025; Bartova et al., 2022; Wang et al., 2020; Guirro et al., 2020; Bian et al., 2025; Liu X. et al., 2024)
Energy	Strong cell-intrinsic pattern involving TCA cycle intermediates, glycolysis metabolites, acylcarnitines, ATP markers, and intracellular acetate	Recurrent changes involving TCA cycle intermediates, ketone bodies, carnitines/ acylcarnitines, glycolysis metabolites, and fermentation metabolites	Broad but heterogeneous changes in TCA cycle intermediates, glycolytic metabolites, ketone bodies, acylcarnitines, ATP markers, and acetate	Host renal, tumor, hepatic, nucleotide-linked, and microbial fermentation patterns; pathogen-only energy collapse findings separated	Recurrent in all model groups, but measurement basis differed	(Sun et al., 2021; Lv et al., 2025; Tang et al., 2024; García-Gavilán et al., 2023; Pomares-Bri et al., 2025; Dai et al., 2024; Messaoudene et al., 2022; Falcone and Rushing, 2025; Wang et al., 2022; Bian et al., 2025; Liu X. et al., 2024)

SCFAs, short-chain fatty acids; TCA, tricarboxylic acid; BCAAs, branched-chain amino acids; AAAs, aromatic amino acids; ADP, adenosine diphosphate. Key supporting studies are representative rather than exhaustive. Pathway-level, modeled, annotation-level, and pathogen-only findings were distinguished from direct host metabolite measurements.

Overall, confidence in the observed patterns differed across the five metabolite themes. For SCFAs, confidence was moderate but qualified because the clearest results came from animal and mixed-model gut studies, particularly where direct fecal measurements were combined with microbiome profiling. Confidence was lower for a consistent direction of SCFA change and for translation to humans because several animal studies had unclear risk of bias and the human results were limited and matrix-dependent. Urolithins were more clearly supported as markers of ellagitannin or ellagic acid exposure and microbial conversion than as general indicators of biological effect. Lipid metabolism was reported across all model groups, but the lipid classes and directions of change varied, so confidence was greater in the broad metabolic pattern than in any individual lipid response. Amino acid and energy metabolism were also reported across model groups, although confidence in a shared response was reduced by differences in study design, sample matrix, analytical method, and risk of bias. Direct metabolite measurements provided a more direct basis for interpretation than modeled, pathway-level, annotation-level, or other indirect results.

## 4 Discussion

### 4.1 Summary of main findings

The main contribution of this review is the distinction between broad pathway distribution and metabolite behavior that varied by model. Lipid, amino acid, and energy metabolism were reported across model groups, whereas SCFAs and urolithins were more closely tied to gut-focused systems and sample matrices. Across the five themes, the specific metabolites, directions of change, and biological interpretation varied with the model system, sample matrix, intervention, analytical platform, and study quality. The risk-of-bias assessment therefore supports greater confidence in broad recurring metabolic processes than in any single metabolite, uniform direction of response, or effect magnitude. The identified patterns should be regarded as candidate signatures that require confirmation in well-designed human studies.

Animal studies most often captured individual and total SCFA responses, but the direction of change varied by model, intervention, and sample type (Table 3) (Fritsch et al., 2022a; Yang et al., 2021; Chen L. et al., 2025; Fritsch et al., 2022b). Studies combining fecal SCFA quantification with microbiome profiling provided clearer links between (poly) phenol exposure, microbial activity, and SCFA production (Table 4) (Alaba et al., 2025; Ávila-Gálvez et al., 2025b; Bian et al., 2025). This aligns with studies showing that anthocyanin-rich interventions can modify SCFA profiles in rodent models (Kapoor et al., 2022). In human studies, the pattern was more limited and less consistent (Table 3) (Lackner et al., 2024; Bartova et al., 2022). This implies that SCFA results may vary depending on the sample matrix, baseline gut microbiota, nutritional context, and whether the study evaluates circulating metabolites, modeled microbial production, or stool concentrations. One possible explanation is that poorly absorbed dietary (poly) phenols reach the colon, where gut microbes can use or transform them in ways that alter microbial fermentation products. This may explain why these metabolites were more evident in gut-focused animal and mixed-model studies, where the gut environment is more directly captured, but limited in isolated cell systems. However, this explanation was not directly tested through standardized comparisons of model systems or sample matrices in the included studies. Accordingly, confidence was moderate but qualified for SCFAs as markers of gut microbial response, with stronger support from animal and mixed-model studies than for a consistent direction of change or translation to humans.

The available studies more directly support urolithins as markers of microbial conversion and exposure than as universal indicators of biological effect (Tsiara et al., 2025; Castellano-Escuder et al., 2022). Their greater detection in human and gut-linked systems may be partly explained by the role of gut microbes in converting ellagitannins and ellagic acid into urolithins (Pinto et al., 2023). This may also explain their absence from isolated cell models, where this conversion cannot occur. Differences in producer metabotypes may also account for variation across individuals and models, although this possibility was not directly examined across the included studies. This is consistent with studies showing that walnut or ellagitannin-rich interventions can increase urinary urolithin A and isourolithin A, and that specific bacterial groups, including *Gordonibacter*, may contribute to urolithin production (Liu H. et al., 2024). One study found that lower levels of systemic inflammatory indicators such as C-peptide, soluble intercellular adhesion molecule-1 (sICAM-1), and soluble interleukin-6 receptor (sIL-6R) were linked to increased urolithin A production (Moussa et al., 2025). Another suggested that responses to polyphenol-rich diets may be stronger among individuals with higher baseline inflammation (Gargari et al., 2025). Together, these observations suggest the possible nutraceutical relevance of urolithins, but they also show that response may depend on baseline physiology, inflammatory status, and gut microbiota composition. The ability of individuals to produce urolithins varies, and producer metabotypes may affect whether diets or supplements high in ellagic acid produce detectable bioactive metabolites (Liu H. et al., 2024; Moussa et al., 2025). Accordingly, the available evidence supports the use of urolithins primarily as markers of ellagitannin or ellagic acid exposure and microbial conversion, rather than as universal indicators of biological effect.

Lipid metabolism emerged as one of the most widely represented themes in the included studies. Unlike SCFAs and urolithins, which varied depending on the model used, lipid metabolites were more consistently reported *in vitro*, animal, human, and mixed-model studies (Figure 3; Tables 2 and 4). However, the specific lipid classes and direction varied by model, disease context, sample matrix, intervention type, and analytical platform. These modifications were most apparent *in vitro* studies and animal models (Table 3) (Sun et al., 2021; Tang et al., 2024; Pomares-Bri et al., 2025; Chen L. et al., 2025). Human studies also reported lipidomic signatures, but these results were broader and more heterogeneous (Table 3) (Chamberlin et al., 2024; Calabrese et al., 2024; Peron et al., 2021; García-Gavilán et al., 2023). In human hepatocytes exposed to lipotoxic stress, metabolites derived from a (poly) phenol blend reduced intracellular triglyceride and cholesterol accumulation, suggesting that (poly) phenol metabolism may influence hepatic lipid handling (Wauquier et al., 2023). In mice, isoflavone intake altered lipid metabolic profiles, including glycerophospholipids and sphingolipids associated with inflammatory status (Shrode et al., 2022). These observations make lipid metabolism a relevant biological context for assessing inflammatory and metabolic responses to dietary (poly) phenols. Lipid responses should not be treated as uniform. A possible explanation for the wider representation of lipid metabolites is that changes in lipids can be measured in many sample types, including cells, tissues, blood, and feces. Furthermore, different lipid groups may also reflect various biological processes. Hence, these changes appeared in the different model groups but differed by class, sample type, disease context, and type of intervention. One study demonstrated that different (poly) phenols induced distinct metabolic perturbation patterns, suggesting that lipid responses may be compound-specific rather than universally applicable to (poly) phenols as a whole (Rushing et al., 2023). This aligns with the review findings, where lipid signatures differed in disease models, tissues, biofluids, and lipid subclasses (Tables 3 and 4). Acylcarnitine shifts appeared *in vitro*, animal, and human studies, including resveratrol-treated cancer cells, curcumin-treated chondrocytes, pulmonary hypertension models, and human dietary interventions (Table 3) (Chamberlin et al., 2024; Sun et al., 2021; Falcone and Rushing, 2025; Deng et al., 2024). These metabolites may reflect fatty acid transport, beta-oxidation, or mitochondrial energy processing; however, their significance varies depending on the specific disease model, tissue type, and direction of change. For instance, metabolites from a (poly) phenol blend coincided with reduced lipid synthesis pathways in one study (Wauquier et al., 2023). Another study suggested that acylcarnitine accumulation may indicate altered coupling between fatty acid oxidation and mitochondrial function (Rushing et al., 2023). Accordingly, confidence was greater in the broad involvement of lipid metabolism across models than in any individual lipid class, metabolite, or direction of change.

Amino acids were reported in all four model groups (Figure 3; Tables 2, 4). However, their direction and significance varied by model, disease context, sample matrix, and whether the metabolites reflected host metabolism, microbial metabolism, or broader inflammatory stress. The inconsistent amino acid patterns could be due to differences in the biological processes examined across studies. These processes might be influenced by factors such as host metabolic stress, tissue injury, or changes in energy demand. Additionally, some variations may arise from amino acid metabolism driven by gut microbes. The most apparent pattern involved tryptophan and indole metabolism. A diet high in (poly) phenols raised circulating indole-3-propionic acid in older persons with intact renal function, indicating that dietary (poly) phenols may affect human host-microbiome amino acid metabolism (Peron et al., 2022). Quinic acid treatment was also associated with modifications in gut microbial tryptophan metabolites, such as indole-3-acetic acid and kynurenic acid, in a metabolic disease model (Li et al., 2024). These findings fit the view that tryptophan-derived metabolites may link dietary (poly) phenols, gut microbial activity, and inflammatory regulation. Microbial indole derivatives can interact with immune and barrier-related pathways, including aryl hydrocarbon receptor signaling, while some tryptophan metabolites have been linked to inflammatory and oxidative stress markers (Peron et al., 2022; Li et al., 2024). Several amino acid shifts may also reflect energy balance, disease state, oxidative stress, tissue injury, or microbial composition. Compared with preclinical studies, human studies showed less consistent changes in amino acid metabolism. For example, a study found that a (poly)phenol-rich diet increased indole-3-propionic acid only in participants with preserved kidney function, suggesting that host physiological status may influence postintervention metabolite changes (Peron et al., 2022). For nutraceutical development, amino acid profiles may help identify host-microbiome responses. Confidence was moderate but qualified, with tryptophan and indole metabolism showing the clearest coherence without forming a universal response marker.

Energy metabolism patterns observed in various models indicate that dietary anti-inflammatory (poly) phenols could impact metabolic stress, mitochondrial function, fatty acid oxidation, and microbial energy pathways. However, biological changes varied; *in vitro* studies illustrated intracellular responses, while human studies predominantly highlighted diverse biofluid profiles (Table 3) (Sun et al., 2021; Casani et al., 2024; Keshteli et al., 2022; Pomares-Bri et al., 2025; Dai et al., 2024; Bartova et al., 2022; Deng et al., 2024; Wu et al., 2022). Nonetheless, these patterns should not be construed as indicating a consistent restorative effect on energy metabolism. Some studies reported movement of metabolites toward control levels, while others showed model-specific or directionally different changes. This could be due to the studies focusing on distinct aspects of cellular energy production rather than a single shared process. Some metabolites might be linked to mitochondrial respiration, while others might stem from glycolysis, fatty acid oxidation, ketone metabolism, or gut microbial fermentation. As a result, the observed changes did not follow a consistent pattern. For instance, in one acute inflammation model, following gallic acid intervention, succinic acid did not exhibit the same trend toward control levels (Table 3) (Wu et al., 2022). This suggests that individual TCA cycle intermediates may hold varying significance depending on tissue type, disease state, and sampling matrix. Mitochondrial function provides biological context for understanding certain metabolic patterns. In a rat fatigue model, resveratrol activated pathways associated with mitochondrial biogenesis and increased citrate synthase and succinate dehydrogenase activities (Lou et al., 2023). This makes it plausible that some TCA cycle and energy metabolite shifts observed in included animal and *in vitro* studies may reflect mitochondrial activity. However, this does not show that all energy metabolism signatures arise from the same mitochondrial pathway. Microbial phenolic metabolites were also associated with changes in urinary metabolic pathways involved in obesity and glucose metabolism, including central carbon metabolism (Chen et al., 2024). This is relevant to the human and mixed-model results in this review, where energy metabolism sometimes overlapped with interactions involving microbial fermentation products (Tables 3, 4) (Bartova et al., 2022; Ávila-Gálvez et al., 2025b; Bian et al., 2025). For nutraceutical development, energy-metabolism profiles may help characterize metabolic stress and fuel use, but confidence was greater in the broad metabolic domain than in a conserved or consistently restorative response.

### 4.2 Cross-model

The available cross-model data did not identify a single conserved metabolomic signature of dietary anti-inflammatory (poly) phenols. Instead, broad metabolic pathways were reported across model groups, while the individual metabolites, directions of change, and biological contexts differed. Lipid, amino acid, and energy metabolism were widely represented in biological systems, while SCFAs and urolithins showed stronger dependence on gut microbial activity, sample matrix, and study design (Figure 3; Tables 2, 4). This distinction is important for nutraceutical research because shared biochemical domains may appear in models even when individual metabolites differ in direction, abundance, or biological significance. A study linked inflammatory biomarkers with changes in fatty acyls, benzenoids, organic acids, and related metabolites, while also showing substantial variation in study design, biospecimen type, and outcome reporting (Carlino et al., 2025). Similarly, studies of dietary (poly) phenol responses, including tea (poly) phenols, suggest that metabolomics can help clarify biological responses in experimental systems, although results may still vary by biological model and omics layer (Willemse et al., 2026). Methodological variation also affected cross-model comparison. For this reason, direct metabolite measurements were separated from pathway-level, modeled, annotation-level, and pathogen-only outputs (Table 4) (Carlino et al., 2025; Willemse et al., 2026). This caution is especially relevant for SCFAs, urolithins, bile acids, acylcarnitines, and TCA cycle intermediates, whose detection depends strongly on analytical workflow and sample type. Overall, metabolomic profiling can help identify exposure biomarkers, response profiles, and priority pathways for nutraceutical development, but these readouts require careful testing before they are treated as stable cross-model markers.

### 4.3 Nutraceutical development

The available studies suggest that metabolomics may help identify candidate exposure and response markers for nutraceutical research. However, the reported metabolite changes have not been shown to predict clinical benefit or translate directly across model systems. Urolithins and their phase II conjugates serve as the more directly supported candidate markers for ellagitannin and ellagic acid metabolism (Table 3) (Domínguez-López et al., 2023; Tsiara et al., 2025; Yaskolka et al., 2023; Castellano-Escuder et al., 2022). SCFAs may reflect gut microbial fermentation after dietary (poly) phenol exposure, although the strongest measurements came from animal and mixed-model studies rather than human interventions (Table 4) (Lackner et al., 2024; Bartova et al., 2022; Ávila-Gálvez et al., 2025b; Bian et al., 2025). Used carefully, these markers may help distinguish dietary exposure, microbial conversion, and downstream metabolic response. Responder profiling is another potential application. Several metabolite groups identified in this review, especially urolithins, SCFAs, and tryptophan-derived indoles, depend partly on gut microbiota composition, host physiology, diet quality, and sample matrix. Individual variation may also impact urolithin production, indole-3-propionic acid responses, and broader metabolic changes after (poly) phenol exposure (Liu H. et al., 2024; Moussa et al., 2025; Peron et al., 2022). Lipid, amino acid, and energy metabolism may provide pathway-level markers of response to dietary (poly) phenol interventions. However, these domains are not specific to (poly) phenols and should not be treated as direct predictors of health benefit. Many of the same metabolites can shift with disease state, stress, diet, tissue injury, or general metabolic adaptation. They should therefore be treated as candidate response markers that require further testing alongside inflammatory biomarkers, dose and formulation data, gut microbiota context, as well as clinically significant outcomes. In this sense, metabolomics can support nutraceutical research by helping to compare formulations, assess bioavailability, identify responsive subgroups, and monitor early biological responses.

### 4.4 Strengths and limitations

A notable strength of this review is its concurrent examination of *in vitro*, animal, human, and mixed-model systems, facilitating comparisons among diverse experimental frameworks (Table 1). The synthesis was organized by model group and metabolite theme rather than pooled without distinction, making it easier to identify where the data were strongest, sparse, or dependent on the model used (Tables 2–4). The review also separated direct metabolite measurements from pathway-level results, modeled outputs, annotation-level features, and pathogen-only responses. This reduced the risk of treating indirect outputs as equivalent to direct host metabolite measurements. By focusing on cross-model distribution rather than isolated metabolite changes, this allowed direct and indirect metabolomic outputs to be interpreted separately in model groups. Several limitations should be acknowledged. In some studies, incomplete reporting of randomization, blinding, missing data, and prespecified analyses limited confidence in individual findings. The included studies differed in intervention type, dose, duration, formulation, study design, disease model, sample matrix, and metabolomics platform. This limited direct comparisons between studies and made quantitative synthesis inappropriate. As a result, the review used narrative synthesis. The distribution of studies was also uneven. Animal studies formed the largest group, while human studies were fewer and more variable (Table 1). Several metabolite themes, especially SCFAs, tissue-level lipid metabolism, and energy metabolism, were more extensively characterized in preclinical models than in humans. Human studies also relied mainly on plasma, serum, urine, stool, or fecal samples, which may not capture tissue-level metabolic changes observed in animal or *in vitro* models. Similar metabolite patterns observed in models should not be interpreted as evidence that these metabolites hold equivalent biological significance in humans. Analytical diversity was another limitation. Differences in platform sensitivity, sample preparation, extraction methods, metabolite coverage, annotation confidence, and statistical pipelines may partly explain why some metabolite classes appeared in some studies but not others. Non-detection should not be treated automatically as biological absence. Some metabolite themes were also captured by different analytical output types. Some results were based on pathway enrichment, modeled flux, annotation-level features, or additional omics outputs rather than direct metabolite-level confirmation. This review separated these data types where possible, but variation in reporting limited the precision of comparisons across studies.

## 5 Conclusion

This systematic review synthesized findings from 78 primary studies examining metabolomic responses to dietary anti-inflammatory (poly) phenols *in vitro*, animal, human, and mixed-model systems. SCFAs were most evident in animal and mixed-model studies focused on gut outcomes, whereas urolithins and ellagic acid-derived metabolites were most prominent in human and mixed-model studies as urinary exposure biomarkers and microbial metabolites. Lipid, amino acid, and energy metabolism appeared more broadly in model groups, although individual metabolites and directions of response varied by model system, sample matrix, intervention type, and analytical platform. These results suggest that metabolomics can help identify candidate biomarkers, pathway-level responses, and model-dependent signatures relevant to nutraceutical development. Variation in study quality reduced confidence in some individual findings, although the recurrence of broader metabolic themes remained evident across model groups. The identified profiles should therefore be treated as candidate signatures rather than direct indicators of clinical benefit. Future research should focus on well-designed human intervention studies that test whether the metabolite changes identified in this review are reproducible and linked to meaningful inflammatory or metabolic outcomes. More consistent methods for sample collection, analysis, metabolite identification, quality control, and reporting would also make studies easier to compare. Studies that use similar (poly) phenol interventions, sampling schedules, and analytical methods across different model systems would provide a stronger basis for judging whether results from experimental models also occur in humans. Future work should also examine how dose, formulation, bioavailability, gut microbiota, and participant characteristics affect metabolite changes.
